# Effect of Fibers on High-Temperature Mechanical Behavior and Microstructure of Reactive Powder Concrete

**DOI:** 10.3390/ma12020329

**Published:** 2019-01-21

**Authors:** Muhammad Abid, Xiaomeng Hou, Wenzhong Zheng, Raja Rizwan Hussain

**Affiliations:** 1 Key Lab of Structures Dynamic Behavior and Control, Ministry of Education, Harbin Institute of Technology, Harbin 150090, China; abidkhg@gmail.com (M.A.); hitwzheng@163.com (W.Z.); 2 Key Lab of the Smart Prevention and Mitigation of Civil Engineering Disasters of the Ministry of Industry and Information Technology, Harbin Institute of Technology, Harbin 150090, China; 3 Civil Engineering Department, College of Engineering, King Saud University, Riyadh 11421, Saudi Arabia; raja386@hotmail.com

**Keywords:** Reactive powder concrete (RPC), fibers, high temperature, mechanical properties, microstructure

## Abstract

This study was aimed to investigate the effect of steel, polypropylene (PP), and hybrid (steel + PP) fibers on high-temperature mechanical properties of reactive powder concrete (RPC). The mechanical properties considered are cubic compressive strength, axial or prismatic compressive strength, split-tensile strength, flexural strength, elastic modulus, peak strain, and stress-strain behavior. The strength recession due to high temperature was investigated at micro level by scanning electron microscope, energy dispersive X-ray spectroscopy, X-ray diffraction, mercury intrusion porosity, thermogravimetric, and differential scanning calorimetry analyses. The high-temperature tests were carried out at target temperatures of 120, 300, 500, 700, and 900 °C. The hot-state compressive strength of RPC started to decrease at 120 °C; however, a partial recovery at 300 °C and a gradual decrease above 300 °C were observed. The degradation of split-tensile strength, flexural strength, and elastic modulus were gradual with increasing temperature despite the effect of different fibers. Whereas, the peak strain was gradually increasing up to 700 °C. However, after 700 °C, it remained unchanged. Steel fiber reinforced RPC (SRPC) and hybrid fiber reinforced RPC (HRPC) showed a ductile behavior. PP fiber reinforced RPC (PRPC) showed a quite brittle behavior up to 300 °C; however, further heating made the microstructure porous and it became ductile too. Overall the performance of SRPC and HRPC were superior to PRPC because of higher modulus of elasticity, higher strength, and better fire resistance of steel fibers. Fiber reinforced RPC was found to have better fire resistance than traditional types of concrete based on comparative studies with the provisions of design codes and earlier research. The constitutive equations developed can be utilized in computer programs for structural design of RPC structures exposed to fire.

## 1. Introduction

Reactive powder concrete (RPC) is the latest generation of concrete having superior properties than traditional types of concrete. It was originally developed in the early 1990’s by Bouygues’ laboratory [1]. The secret of its high strength, remarkable durability, and excellent toughness lie in its dense microstructure and use of fibers [2,3]. Furthermore, the use of high amount of cement content, elimination of coarse aggregates, addition of reactive quartz sand, reducing the water to binder (w/b) ratio to less than 0.2, addition of pozzolanic materials, and addition of microfibers make it superior to others [4,5]. It is primarily used as an ultra-high performance concrete (UHPC) based on its superior mechanical properties, low permeability, and excellent durability. The demand for RPC has increased over the last two decades, especially in the precast concrete production industry. Furthermore, it is being used in buildings, bridges, tunnels, and nuclear structures [6,7].

The use of high amount of cement in RPC has adverse effect on its early age shrinkage and hardened performance. This problem has been resolved by using mineral additives such as fly ash, silica fume, and slag [8]. The fresh and hardened properties of concrete were modified and improved by replacing some percentage of cement with these mineral additives [9]. Moreover, the reuse of these industrial by-products will contribute to environmentally safe green construction and will minimize their dumping and hazardous problems in the environment [10,11]. 

With the advancement in technology, the risk of fire is increased in infrastructure and much attention is being required to fire safety design to minimize the structural damage and loss of lives during accidental fires. For instance, the building of School of Architecture, Delft University of Technology, Netherland collapsed during an accidental fire, although the occupants were evacuated from the building. The main reason for structural collapse was assumed to be the fire-induced spalling which reduced the load bearing capacity of structural members [12]. On the other hand, some structures are designed to bear high temperatures such as nuclear structures and industrial structures. Furthermore, some structures are exposed to thermal fatigue such as parking apron of airports [13]. Therefore, structures should be designed carefully to withstand high temperature.

The fire resistance of concrete structures is dealt with many regulation and standards in different parts of the world. Among them, The Eurocode-2 [14], ACI code [15], ASCE manual for structural fire protection [16], and Concrete Association of Finland (RakMK) guidelines [17] are important documents. However, the scope of these design codes is limited to traditional concrete types and the relatively new type of materials including RPC are not covered in it. However, in the last decade, extensive studies were reported for RPC and UHPC, especially at ambient temperature [18,19,20,21]. In spite of a promising material at ambient temperature, fire-induced spalling limits its application to buildings and the current design codes allows us to use only up to Class C90/105 for concrete structures [14,22,23]. Recently, several studies found out that the incorporation of proper dosage of polypropylene (PP) fibers, steel fibers, and hybrid combination (PP + steel) of fibers prevents explosive spalling [24,25,26]. PP fibers leave micro-channels after melting at nearly 167 °C, through which the trapped vapors could be released resulting in a decrease in the vapor pressure [26]. Conversely, the steel fibers increase the tensile capacity of concrete and thus the resistance against fire-induced spalling increases [27]. These beneficial effects made it possible to use RPC under high temperature as well.

In the last decade, extensive fire-resistance studies of RPC were mainly carried out at ambient condition after exposure to high temperature [7,24,26,27,28,29,30,31,32,33,34,35,36]. However, there are fewer studies exist which cover RPC under hot-state as well [37,38]. But the study regarding the effect of different types of fibers on the mechanical properties of RPC at high temperature is scarce. Furthermore, the split-tensile strength, flexural strength, elastic modulus, and stress-strain behavior studies were limited at high temperature. The detailed investigations regarding the behavior changes of RPC due to high temperature at microstructure level are missing. The aim of this study is to evaluate the mechanical properties of steel fiber reinforced RPC (SRPC), PP fiber reinforced RPC (PRPC), and hybrid fiber reinforced RPC (HRPC) as well as their associated microstructural changes due to high-temperature exposure. Mechanical properties consist of cubic and prismatic compressive strength, split-tensile strength, flexural strength, elastic modulus, stress-strain behavior, and peak strain. The changes of mechanical properties of RPC are correlated with the microstructural and chemical changes at high temperatures. Hence, the microstructure investigation by means of mercury intrusion porosity (MIP) measurements of the pore characteristics, scanning electron microscopy (SEM) analysis for studying micrographs, energy dispersive **X**-ray spectroscopy (EDX) to know the chemical composition and atomic weight of chemical hydrates, thermogravimetric (TG) and differential scanning calorimetry (DSC) for determination of hydration reaction, and **X-**ray diffraction (XRD) analysis for crystalline composition of RPC matrix was performed in this study. The study was carried out at 6 target temperatures of 20, 120, 300, 500, 700, and 900 °C. The results obtained for mechanical properties at high temperature were compared with the same properties obtained from the design codes and published literature. Moreover, simplified equations were developed from the experimental results. These predictive equations can be used in computer programs for fire resistant design of RPC structures.

## 2. Experimentation

### 2.1. Materials and Mix Proportions

Portland cement, silica fume, slag, quartz sand, superplasticizer, steel fibers, and PP fibers were the main ingredients for the preparation of RPC. The main properties of various constituents are as follow:

#### 2.1.1. Cement

Ordinary Portland cement (P.O 42.5) was provided by Yatai Cement Co., Ltd. (Jilin, China). The quality assurance was controlled as per the Chinese specifications for ordinary Portland cement [39]. It was ensured to use the open cement bag within 1 month. The chemical compositions provided by the manufacturer are shown in Table 1.

#### 2.1.2. Silica Fume

Silica fume is an ultra-fine powder of dark grey color. It was provided by Jinshi Building Material Company, Gongyi City, Henan Province, China. The average particle size was 0.1–0.3 μm, SiO_2_ content was 94%, and the bulk density was 1700 kg/m^3^. The chemical composition provided by the manufacturer is shown in Table 1.

#### 2.1.3. Slag

The slag produced by Harbin Sanfa New Energy Building Materials Co., Ltd. (Harbin, China). was used. It is an off-white powder. Its density, specific surface area and 28 days activity index was 2.85 g/cm^3^, 366 m^2^/kg, and 103%, respectively. The chemical composition provided by the manufacturer is given in Table 1.

#### 2.1.4. Quartz Sand

The quartz sand was supplied by Harbin Jinghua Water Treatment Material Co., Ltd. (Harbin, China). Fine and coarse quartz sand were used with equal ratios. The average nominal size of coarse sand was 0.4 mm and fine sand was 0.2 mm. The SiO_2_ content in quartz sand was more than 99.6%.

#### 2.1.5. Polycarboxylate Superplasticizer

The latest generation polycarboxylate superplasticizer produced by Qingdao Hongxia Concrete Water Reducing Agent Co., Ltd. (Qingdao, China). was used. The PH value was 6~8, water reduction rate was 25%–35%, specific gravity was 1.08 g/cm^3^, and the solids content was 40%.

#### 2.1.6. Steel Fiber

The straight brass coated steel fiber produced by Changhong Company, Anshan, Liaoning Province was used. The average length was 13 mm, the average diameter was 0.22 mm, the tensile strength was 2850 MPa, and the elastic modulus was 200 GPa.

#### 2.1.7. Polypropylene Fiber

High-strength polypropylene fiber having a tensile modulus of 3.5 GPa and a tensile strength of 360 MPa was used. It is chemically inert and has low fire resistance with a melting point of 167 °C. It was provided by Ruixin Fiber Factory, Shijiazhuang, Hebei province. The length was 18–20 mm, average diameter was 45 μm, and density was 0.91 g/cm^3^.

The mix proportion was optimized through basic trial experiments and the effectiveness of the raw materials was confirmed by comparing the 7 days strength with the earlier studies [29,34,38]. The weight ratios of cement: silica fume: slag: quartz: and superplasticizer are 1:0.3:0.15:1.2:0.04. The water to binder (w/b) ratio is 0.16. Based on the optimum mix ratios, through mixing different volume dosages of steel and PP fibers, three RPCs (SRPC, PRPC, and HRPC) were produced in this study. The corresponding steel fiber and PP fiber volume dosages for the three RPCs were (2%, 0%), (0%, 0.3%), and (2%, 0.2%), respectively. The mix proportions used in this study are summarized in Table 2. PRPC is considered as control/reference concrete because PP fibers melt above 167 °C and it becomes as plain RPC. Furthermore, comparisons with the plain RPC [40], traditional types of concrete, and design codes or recommendations were carried out in this study. During the preliminary trial experiments, it was ensured that the amount of fiber dosage was effective to prevent fire-induced spalling during coupled thermo-mechanical loading.

### 2.2. Specimens Fabrication and Curing

The cubic compressive strength and split-tensile strength tests were carried out on the cubic samples of size 70.7 mm × 70.7 mm × 70.7 mm. While for axial or prismatic compressive strength, flexural strength, elastic modulus, and stress-strain curves, the prismatic specimens of size 70.7 mm × 70.7 mm × 220 mm were used. A total of 270 specimens were prepared out of which the cubic and prismatic specimens are 108 and 162, respectively. The mechanical properties for each RPC were determined at six target temperatures (20, 120, 300, 500, 700, and 900 °C) and for each target temperature, three specimens were used. However, the average of a pair of tests was reported for each target temperature. Furthermore, if the deviation among the two tests results was more than 5% then a third sample was tested and the deviated test was discarded [41]. Therefore, the total number of specimens tested for each type of mechanical property (cubic compressive strength, axial or prismatic compressive strength, split-tensile strength, flexural strength, and stress-strain behavior) is approximately 45, considering the repetitive third test conducted sometimes when the deviation was more than 5%.

The specified proportions of the materials for the RPC mixes were measured with an electronic balance. A horizontal pan-type of mixer was used for mixing. The mixer can be adjusted for revolution at different speeds. Initially, the dry materials were mixed for 3 min at a slow speed of 140 ± 10 revolutions/min. The top opening was properly covered with the moving cover to prevent the materials from dispersion into the air. Water and liquid superplasticizer were poured into the properly mixed dry materials and stirred at high speed of 280 ± 10 revolutions/min for 5 min. A uniform plastic consistency mix was produced. In the last stage, fibers were sprinkled in rotating wet concrete over the course of 2 min and mixed for additional 5 min. The entire mixing process took about 15 min. The RPC was quite flowable and the workability was tested for each mix using mortar flow table test method as per the relevant Chinese standard [42]. A mini-slump cone in the middle of a vibratory table was filled with fresh RPC. The cone was removed and RPC was first allowed to spread naturally on the table, then the flow table was dropped 25 times. The workability of RPC was determined by measuring the spread diameter of the RPC. The measured flowability of SRPC, PRPC, and HRPC was 175, 180, and 170 mm, respectively.

Immediately after measuring the workability, RPC was used to prepare the hardened specimens. Plastic molds were properly cleaned and oiled before using. Each mold was filled in 3 layers and properly compacted on a vibratory table until the surface covered with oozing slurry. The 3rd layer was smoothed with a trowel after compaction. The molded specimens were stored in laboratory conditions at 25 °C and 70% relative humidity for one day. On the following day, the specimens were demolded and cured for 72 h in an accelerated steam curing box with a water temperature of 90 °C. Steam curing makes the RPC stronger by accelerating the hydration reactions. The specimens were then stored in laboratory conditions for 60 days.

From the pioneer’s investigations, it was found that RPC is very compact and the moisture must be removed through heating process in order to reduce the explosive spalling risk [7,29]. The specimens were stored at 105 °C in the oven. The moisture content of RPC was lowered up to 2%–3.5% of its initial wet weight. The normal duration was 5 to 7 days for this heat treatment process. The average rate for moisture loss was 0.71% per day. In order to protect the specimens from moister absorption, it was preserved in sealed polyethylene bags until the day of testing.

### 2.3. Testing Approach

#### 2.3.1. High-Temperature Tests Equipment

**Figure 1:**
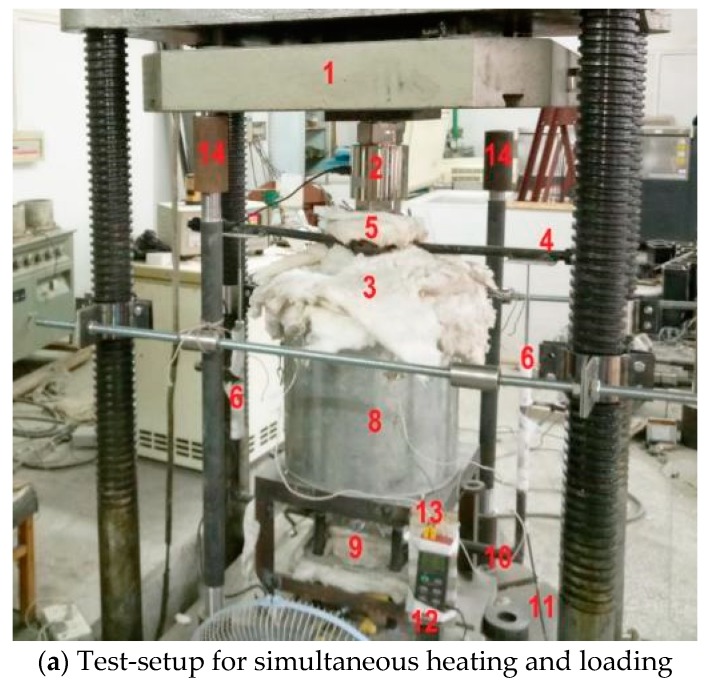

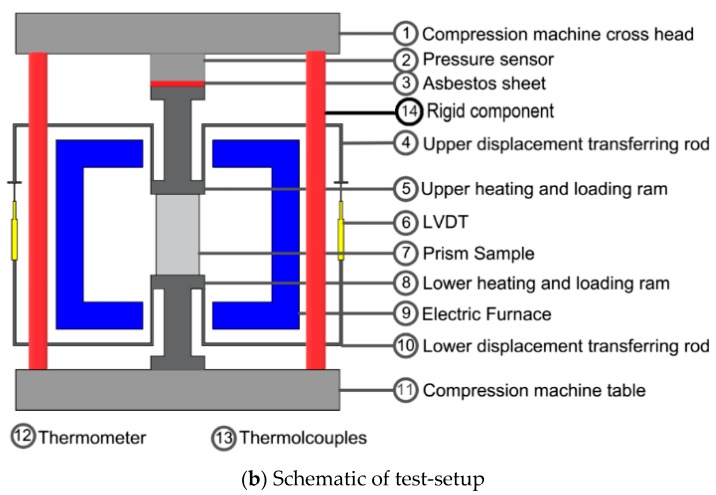
Experimental apparatus used in the study.

The high-temperature tests were conducted in a purpose-built furnace having openings on upper and lower side, which facilitate the simultaneous loading during the heating process. The furnace was 400 mm tall, 400 mm external diameter, and 250 mm internal diameter. The test-setup frame and furnace arrangements are shown in Figure 1a, whereas the schematic representation is shown in Figure 1b. The loading from the crossheads of the loading machine was transferred to the testing arrangement through special high-temperature resistant alloy platens.

The openings of the furnace were covered with a ceramic sheet to limit the heat loss. Furthermore, the exposed part of the loading platens was wrapped with ceramic sheets as a safety precaution. The length of alloy platen also works as an insulator and protects the testing frame and equipment from extreme heating. The furnace temperature was measured with a K-type thermocouple, mounted at the middle of the furnace. The furnace can be heated up to 1200 °C with a maximum heating rate of 30 °C/min. Load was transmitted by a 100-ton computer controlled universal testing machine (UTM) at a different loading rate, depending on test requirements. A 100-ton pressure sensor was also used to record the loading history. The specimen deformation was measured by linear variable differential transformers (LVDTs). The test data were recorded with a “WS3811-Beijing Wave Spectrum Data-logger”. A special steel frame was bolted to the UTM to hold the upper loading platen after the failure of the specimen (free fall of upper loading platen) and protect LVDTs and furnace arrangements from damaging. 

**Figure 2:**
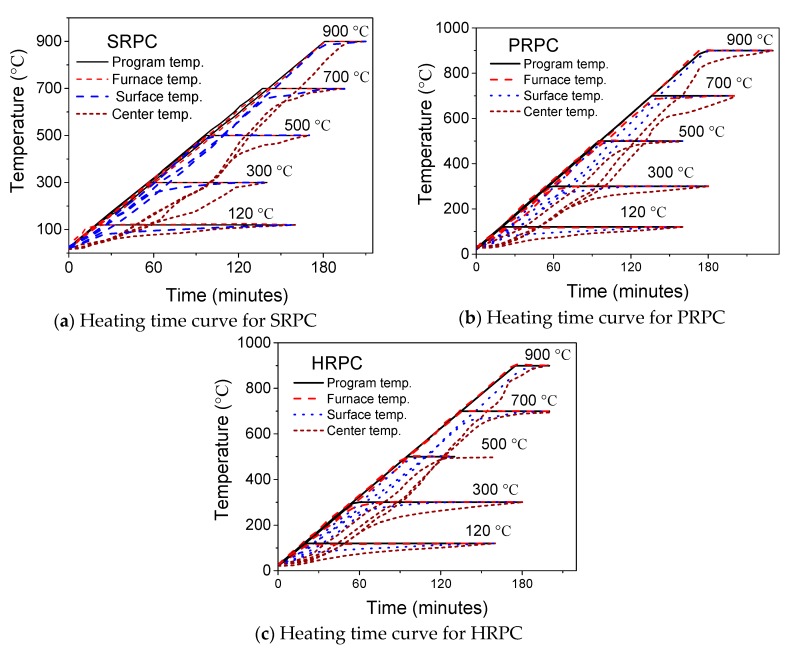
Heating-time curve for high-temperature mechanical testing.

The central temperature of the prismatic specimens was measured during compressive strength testing. A thermocouple was installed in the center core of the specimen at the midpoint location during the specimen fabrication process. Two thermocouples were attached to the surface on the longitudinal midpoint location at the diametric opposite directions. It was ensured during the whole testing process that the surface thermocouples kept in touch with the specimen. Center-309 thermometer programme was used to record the temperature variation after 2 min interval. WRNK-0101 type thermocouples of two sizes were used. The specimen center temperature was recorded by 0.5 mm flexible thermocouple, whereas the surface temperature was measured by 3 mm rigid thermocouple. Both thermocouples are capable of measuring the temperature up to 1000 °C, with a possible error of ± 2.2 °C. The heating procedure followed in this study was according to the recommendations of RILEM [43,44] for high-temperature strength evaluation of concrete in service and accidental conditions. The temperature range for service condition is from 20 °C to 200 °C, whereas for accidental conditions the temperature range is from 20 °C to 750 °C. However, the structural tests are usually conducted for more realistic fire exposures where temperature can be higher than 75 °C [45]. Therefore, the upper limit selected for the heating regime was chosen as 900 °C. In order to properly capture the effect of phase changes in concrete due to heating, the high-temperature tests were carried out at small temperature intervals [46]. The target temperature points selected are 20, 120, 300, 500, 700, and 900 °C. This heating regime comprising most critical temperature points was used, keeping the physical and thermal changes in RPC mix and the spalling boundary conditions (300–500 °C) in mind. The specimens were heated until the center temperature reached the target temperature. The heating-time curves for SRPC, PRPC, and HRPC specimens are shown in Figure 2. It is evident that the gradient of surface temperature and the center temperature is lower than the furnace and programme temperature. 

The specimen deformation has been transferred to the ambient condition by the attached alloy rods. The alloy rods were welded only with those ends of the platens which are connected with the specimen. The special “L” shape alloy rods were welded with the bottom of the top alloy platen in the diametric opposite directions. Similarly, two “U” shape alloy rods of the same material as alloy attachment were welded to the top of the bottom platen. LVDTs were attached with the outside leg of “U” shape bottom rods. Thus the relative deformation of the specimen can be measured by averaging the deformation of two LVDTs. Since the deformation was measured on the diametric opposite directions, the eccentric loading and non-rigid loading ram effects were removed.

#### 2.3.2. Mechanical Properties Tests

**Figure 3:**
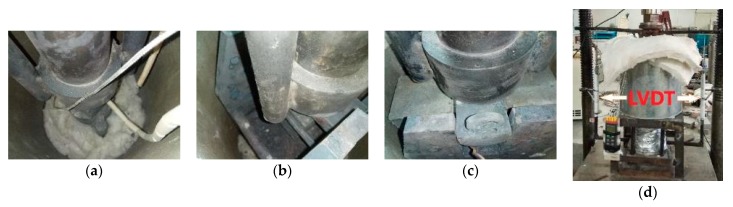
Experimental setup for hot-strength tests: (**a**) compressive strength test, (**b**) split-tensile strength test, (**c**) flexural strength test, and (**d**) elastic modulus test.

Compressive strength, splitting tensile strength, flexural strength, and elastic modulus tests were measured in accordance with Chinese Standard GB/T 50081 [47]. These tests were performed on computer controlled universal testing machine (UTM) of 1000 kN capacity. The apparatus for the hot-strength test are shown in Figure 3. A circular electric furnace, as depicted in Section 2.3.1, was used, inside which a sample was placed between the alloy platens for compression, elastic modulus, and stress-strain tests. The alloy platens transfer load from the lower and upper crosshead of the compression machine. The alloy platens also transfer heat to the pressed specimen surface. For split-tensile strength and flexural strength tests, a special custom-made fixture was inserted inside the furnace. The high-temperature tests can be carried out by one of the following three methods; stressed tests, unstressed tests, and unstressed residual property tests [43,44,48]. In the unstressed tests, the specimens are loaded till failure after the target high temperature is achieved. In stressed test, a pre-load (10% to 40% of the room temperature compressive strength) is applied before the heating and sustained during the heating. The specimen is crushed once the target temperature is achieved. Whereas in the unstressed residual test, the specimen is not loaded during the heating process. After heating, the specimen is allowed to cool down to room temperature and then it is loaded until failure. In this study, the high-temperature unstressed procedure was adopted. The loading rate for compressive strength and elastic modulus tests was 0.3 mm/min. For split-tensile strength and flexural strength tests, the loading rate was 0.05 mm/min. The stress-strain curve was determined at the unstressed condition. The loading rate used for the stress-strain curve was 0.1 mm/min [29]. The stress-strain curve consists of two branches, initially an ascending branch up to the peak stress and then a descending branch. The ascending branch is measured easily as compared to the descending branch. The specimen and loading machine interaction makes it difficult to measure the descending branch. When the specimen is loaded, the resultant deformation accumulates the strain energy in the specimen and loading machine. Once the specimen reaches its peak deformation, the strain energy stored in the machine suddenly releases and causes a failure of the specimen. This problem becomes more prominent when the specimens possess high compressive strength. Since RPC have superior strength, this problem was evident in the stress-strain testing procedure. Such problem can be resolved by inserting a steel column in parallel with the specimen and to lock it also along with the specimen just before the ultimate stress. By this way, the strain energy stored will not be released by the failure of the specimen and the complete stress-strain curve can be measured [49,50]. To define the complete stress-strain curves of RPC at elevated temperatures, the final compressive strain and the total stress-strain curves in this study were developed using high strength alloy steel rigid components. The test arrangement along with a schematic diagram is shown in Figure 1. The cross-sectional area and length of the specimens were measured using a micrometer. Furthermore, the slope of the stress-strain curve is used for determination of secant elastic modulus. The split-tensile strength and flexural strength were measured as per Equations (1) and (2), respectively.

(1) $$f_{t}=\frac{2F}{\pi A}=0.637\frac{F}{A}$$

where *F* is the failure force in *N* and *A* is the area of split face in mm^2^. The resultant split-tensile strength is in MPa.

(2) $$f_{f}=\frac{1.5FL}{b^{3}}$$

where $f_{f}$ (MPa) is flexural strength, *F* (N) is the failure force applied at the midpoint of the specimen, *L* (mm) is the distance between the supporting rollers in custom-made fixture, and b (mm) is the lateral dimension of the cross-section.

The experimental results were modeled using linear and nonlinear regression analyses in commercially available software “OriginPro”. The mechanical properties were used as response parameter and the temperature as the predictor parameter. The accuracy of analysis is measured by the coefficient of determination “*R*^2^”, that shows the sum of the square of deviations of the response values to their predictor parameter. The variation of *R*^2^ is from 0 to 1, where 1 shows the best-fitted model [51]. The R^2^ values for our proposed models lie in between 0.85 to 0.99. This shows a reasonably high confidence level considering the varying nature of concrete in term of its mechanical performance.

#### 2.3.3. Microstructure Tests

The microstructure changes in the RPC specimens after exposure to the target temperatures were investigated by SEM, EDX, XRD, and MIP tests. In contrast, the primary hydration reactions during exposure to high temperature were investigated by TG and DSC analyses. 

The cubic specimens after the high-temperature tests were crushed and the specimens of about 5 mm diameter in the form of pellet shape were prepared for SEM and EDX analyses. The specimens were immediately soaked in acetone liquid to stop the additional hydration reactions. The specimens were dried in an oven at 60 °C, in order to attain a constant dry weight. Furthermore, to make the specimens conductive, they were gold-sprayed. Then the specimens were photographed by Quantum 200 scanning electron microscope. The microstructure and chemical composition of RPC matrix, the bonding interface between PP fiber and matrix, and the bonding interface between steel fiber and matrix were mainly investigated.

The MIP test was conducted to measure the porosity and pore-size distribution of the specimens at the target high temperatures. The boundary condition of pressure and pore sizes of MIP equipment is 0.01–228 MPa and 0.0036–1000 μm, respectively. The specimens of a random shape of approximately 5 mm size were extracted from the crushed cubic sample after the high-temperature test. The specimens were dried in an oven at 60 °C for 8 h. 

The phase composition study of RPC specimen after exposure to heating regimes was conducted on finely ground powder using X-ray diffraction method. The cubic specimens were passed through the same target temperatures and then crushed to obtain the small pieces. The crushed pieces were finely ground into powder which was dried at 60 °C for 8 h. The scanning speed of XRD was 5°/min, and the range of the diffraction angle was 10~80°. The result was analyzed and the different changes in the microstructure were investigated. 

The different hydration reactions of RPC matrix were studied by TG and DSC tests using STA449F3 and DSC200F3 equipment. The 20–30 mg sample of finely ground powder obtained from the inner core of cubic specimen was prepared for each test. The powder samples were heated from room temperature to 1000 °C, using a heating rate of 5 °C/min.

## 3. Test Results and Discussion

### 3.1. Mechanical Properties

#### 3.1.1. Compressive Strength

**Figure 4:**
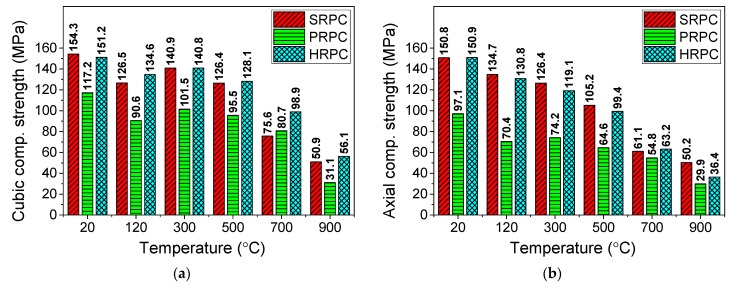
Compressive strength of RPC for (**a**) cubic and (**b**) prismatic specimens.

The failure loads of cubic and prismatic specimens were used to compute the compressive strengths of different RPC mixes, which are shown in Figure 4. The high temperature results in significant physical and chemical changes and decreases the compressive strength of RPC. The compressive strength of PRPC is significantly lower than those of SRPC and HRPC due to the lower strength and elastic modulus of PP fibers as compared to steel fibers. The compressive strength started to decrease at 120 °C, however at 300 °C, a partial recovery was seen for all types of RPC. The initial decrease in compressive strength is attributed to the coupled effect of build-up internal vapor pressure and loading [13,52]. The expansion of water between the C-S-H gel layers also reduced the binding forces [53]. The strength recovery at 300 °C was mainly due to the increase in Van der Walls forces (surface forces) due to the removal of free water [54,55]. The porosity of the concrete has a direct effect on strength when free water is removed [56]. Above 300 °C, a gradual decrease in cubic and a shape decrease in prismatic strength were observed. The decrease in strength was attributed to various factors which are explained in the microstructure analysis section. The decomposition of C-S-H and CH hydrates in the range of 400–600 °C resulted in a decrease in the strength. Further reduction was obtained due to the phase transformation of quartz aggregate from α to β phase at 573 °C [57]. Furthermore, above 700 °C, the bonds between cement past, aggregate, and steel fibers were severely deteriorated due to uneven expansion among them. The severe cracking at 900 °C resulted in more than 70% strength loss. 

**Figure 5:**
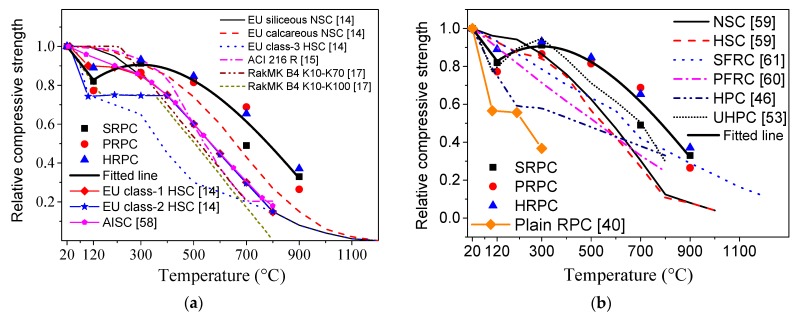
Comparison of the normalized compressive strength of reactive powder concrete (RPC) with (**a**) design codes and (**b**) previous research.

The normalized compressive strength has been compared with the strength obtained from different design codes [14,15,16,17,58] in Figure 5a. The compressive strength below 300 °C is lower than those obtained from the design codes. This is again attributed to the trapped vapor pressure under the loading effect, which is not seen in the cases of NSC and HSC because of high porosity. However, above 300 °C, the strength of RPC is much higher than those given by the design codes. This confirms that the superior microstructure results in a better compressive strength at higher temperature. 

The normalized compressive strength of different fiber-reinforced RPCs has also been compared in Figure 5b with the computed/experimental strength values of other concretes obtained from several studies as follow:

- The Aslani and Bastami [59] fitting model for normal strength concrete (NSC) and high strength concrete (HSC) with no fiber content.
- The Aslani and Samali [60] fitting model for PP fiber-reinforced normal strength concrete (PFRC).
- The Aslani and Samali [61] fitting model for steel fiber-reinforced normal strength concrete (SFRC).
- The Khaliq and Kodur [46] fitting model for plain high performance concrete (HPC), steel fiber-reinforced high performance concrete (SHPC), PP fiber-reinforced high performance concrete (PHPC), and hybrid fiber-reinforced high performance concrete (HHPC).
- The Xiong and Liew [53] experimental results of ultra-high strength concrete (UHSC).
- The Zheng et al. [40] experimental results of Plain RPC.

The present study is in good agreement with the results of UHSC as reported by Xiong and Liew [53]. The normalized pattern is again similar to those of the design codes. The degradation in strength was more than the aforementioned concrete types except HPC [46] and UHSC [53] up to 300 °C. However, above 300 °C, RPC performs better than other traditional types of concrete [59,60,61] due to its superior microstructure and effective role of fibers to resist spalling. It is also evident from Figure 5b, that the degradation in Plain RPC [40] is severe and more than those of all other concrete types up to 300 °C. It might be attributed to the severe effect of vapor which was trapped in plain RPC [40] and caused severe degradation when coupled with compressive loading. On the other hand, PP fibers melted at nearly 167 °C and released the vapor pressure by providing micro-channels and thus improving the compressive strength. Whereas steel fibers, due to their effective role against crack propagation at high temperatures, resulted in improved compressive strength.

The experimental results were modeled by regression tool to propose mathematical equation. The formula for normalized cubic and axial or prismatic compressive strength are given in Equations (3) and (4), respectively.

(3) $$f_{cu}^{T}/f_{cu}=\{\begin{array}{ll} 1.03-0.00172T, & 20\;{}^{\circ}C\leq T\leq 120\;{}^{\circ}C\\ 0.68+0.0016T-3.16\times 10^{-6}T^{2}+1.03\times 10^{-9}T^{3}, & \;120\;{}^{\circ}C<T\leq 120\;{}^{\circ}C \end{array}$$

(4) $$f_{c}^{T}/f_{c}=1.018-7.82\times 10^{-4}T,\;20\;{}^{\circ}C\leq T\leq 900\;{}^{\circ}C$$

where $f_{cu}^{T}/f_{cu}$ is the normalized cubic compressive strength, $f_{c}^{T}/f_{c}$ is the normalized axial or prismatic compressive strength, and *T* is the temperature in degree Celsius.

#### 3.1.2. Split-Tensile Strength

**Figure 6:**
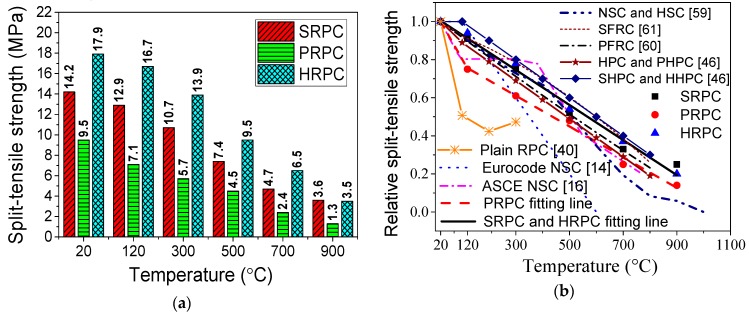
Split-tensile strength of RPC: (**a**) Absolute values and (**b**) comparison with earlier research and design codes.

The importance of tensile strength under high temperature is vital. Unlike the ambient temperature studies, it cannot be ignored in high-temperature conditions [62]. Tensile strength resists the crack propagation and, furthermore, it safeguards concrete against build-up vapor pressure at high temperature and resists fire-induced spalling [63,64,65]. The evolution of split-tensile strength of SRPC, PRPC, and HRPC with high temperature is shown in Figure 6a. The split-tensile strength of SRPC, PRPC, and HRPC at ambient temperature is 14.2, 9.5 and 17.9 MPa, respectively. The split-tensile strength of PRPC is much lower than the others due to lower elastic modulus and small volume ratio as compared to the steel fibers. Furthermore, the HRPC split-tensile strength is higher than the SRPC due to the additional resistance provided by the PP fibers against the tension force. The degradation of split-tensile strength for all types of RPC is gradual with increasing temperature. This is because of the same reasons as explained in Section 3.1.1. The split-tensile strength of SRPC, PRPC, and HRPC at 500 °C was reduced to 52%, 47%, and 53% of its room temperature strength, respectively. The percentage of split-tensile strength remaining at 900 °C for SRPC, PRPC, and HRPC was 25%, 14%, and 20%, respectively. The ratio of the split-tensile strength of RPC at high temperature to that at room temperature is plotted in Figure 6b. The split-tensile strength values obtained from Eurocode-2 [14], ASCE’s structural fire protection committee report on NSC [16], and the earlier research [46,59,60,61] have also been compared with the experimental results of this study. The PRPC have very low tensile strength when compared to NSC [59], HSC [59], SFRC [61], PFRC [60], SHPC [46], HHPC [46], and NSC (ASCE) [16]. However, its performance is better than Eurocode-2 [14] NSC above 300 °C. The SRPC and HRPC have similar trends to the SHPC [46] and HHPC [46]; however, they are more conservative than the other types of concrete [59,60,61]. The plain RPC [40] not only suffered from explosive spalling above 300 °C but also has the worst performance in terms of tensile strength among all types of concretes. This is again attributed to the coupled effect of vapor and loading, which causes severe deterioration.

The experimental results were modeled by regression tool to propose mathematical equation. The formula for the normalized split-tensile strength of SRPC and HRPC is given in Equation (5), whereas that for PRPC is given in Equation (6).

(5) $$f_{t}^{T}/f_{t}=1.022-9.21\times 10^{-4}T,\;20\;{}^{\circ}C\leq T\leq 900\;{}^{\circ}C$$

(6) $$f_{t}^{T}/f_{t}=\{\begin{array}{ll} 1.05-0.00247T, & 20\;{}^{\circ}C\leq T\leq 120\;{}^{\circ}C\\ 0.85-8.06\times 10^{-4}T, & \;120\;{}^{\circ}C<T\leq 900\;{}^{\circ}C \end{array}$$

where $f_{t}^{T}/f_{t}$ is the normalized split-tensile strength and *T* is the temperature in degree Celsius. 

#### 3.1.3. Flexural Strength

**Figure 7:**
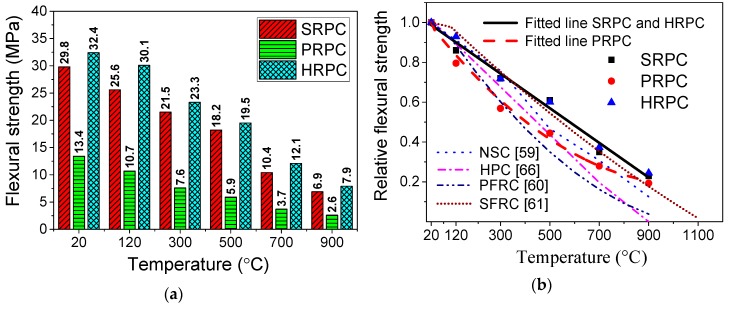
Flexural strength of RPC: (**a**) Absolute values and (**b**) comparison with earlier research.

The absolute and normalized flexural strength of RPCs at different target temperatures are plotted in Figure 7. The flexural strength of SRPC, PRPC, and HRPC at room temperature is 29.8, 13.5, and 32.4 MPa, respectively. This is consistent with the split-tensile strength results and the incorporation of steel fibers increased the flexural strength as compared to PP fibers alone. The flexural strength of HRPC is maximum because of the additional resistance provided by the PP fibers against crack propagation. The degradation of the flexural strength was linear for SRPC and HRPC. In contrast, it was parabolic for PRPC. The flexural strength of SRPC, PRPC, and HRPC was reduced to 23%, 19%, and 24% of its room temperature strength, respectively, at 900 °C. The behavior of PRPC was very brittle after the melting of PP fibers at and above 300 °C, whereas the SRPC and HRPC showed a ductile behavior until 900 °C.

The flexural strength of RPC has been compared with those of NSC [59], PFRC [60], SFRC [61], and HPC [66] in Figure 7b. From the comparison, it is evident that PRPC performance is poor and somehow similar to PFRC [60] up to 500 °C; however, above 500 °C, a little improvement was observed. The decrease in the flexural strength of SRPC and HRPC was relatively small as compared to the traditional types of concrete [59,60,61,66] due to the combined effect of superior microstructure and steel fibers. PP fibers have a little contribution due to melting at 167 °C.

Using regression analysis, the relationship of the normalized flexural strength ($f_{f}^{T}/f_{f}$) with temperature can be expressed by the following equations;

For SRPC and HRPC;

(7) $$f_{f}^{T}/f_{f}=1.004-8.69\times 10^{-4}T,\;20\;{}^{\circ}C\leq T\leq 900\;{}^{\circ}C$$

For PRPC;

(8) $$f_{f}^{T}/f_{f}=1.001-0.00156T+7.48\times 10^{-7}T^{2},\;20\;{}^{\circ}C\leq T\leq 900\;{}^{\circ}C$$

where $f_{f}^{T}$ is the flexural strength at high temperature, $f_{f}$ is the flexural strength at room temperature, and *T* is temperature.

#### 3.1.4. Elastic Modulus

**Figure 8:**
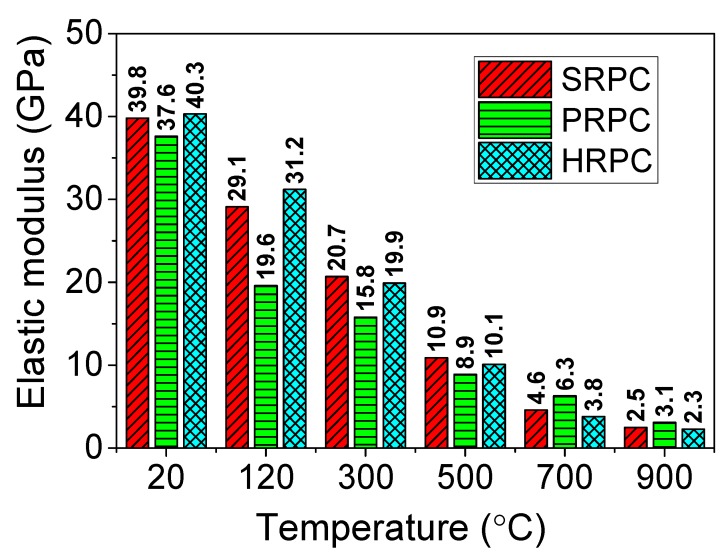
Elastic modulus of RPC as a function of temperature.

The evolution of elastic modulus of RPC with temperature is shown in Figure 8. The trends show that severe degradation was observed for all types of RPC. Especially, PRPC has a large drop in elastic modulus at 120 °C. The unheated elastic modulus of SRPC, PRPC, and HRPC are 39.8, 37.6, and 40.3 GPa, respectively. It can be seen again that the effect of fibers is consistent with the earlier results. Unlike the compressive, split-tensile, and flexural strengths, the reduction in elastic modulus was severe and almost 50% reduction was observed at 300 °C for all types of RPC. The reduced elastic modulus of SRPC, PRPC, and HRPC at 900 °C was 2.5, 3.1, and 2.3 GPa, respectively. This is 7%, 9%, and 6% of its respective original unheated value. The elastic modulus was affected by the same factors which affected the compressive strength [67]. However, the degradation in elastic modulus is more severe that observed in the case of compressive strength. This might be attributed to various factors such as physical and chemical changes (trapped vapor; decomposition of Ca(OH)_2_, C–S–H and CH hydrates), cracking of interfacial transition zone (ITZ), and increasing thermal incompatibilities between aggregate, paste, and steel fibers [68]. The elastic modulus of PRPC is marginally higher than SRPC and HRPC at 700 and 900 °C. This can be attributed to the additional deterioration due to thermal incompatibilities between steel fibers and RPC matrix at such high temperatures. Furthermore, the porous volume in PRPC increases due to melting of PP fibers, which may reduce the thermal incompatibilities between the aggregate and paste owing to an increase in free space [69].

**Figure 9:**
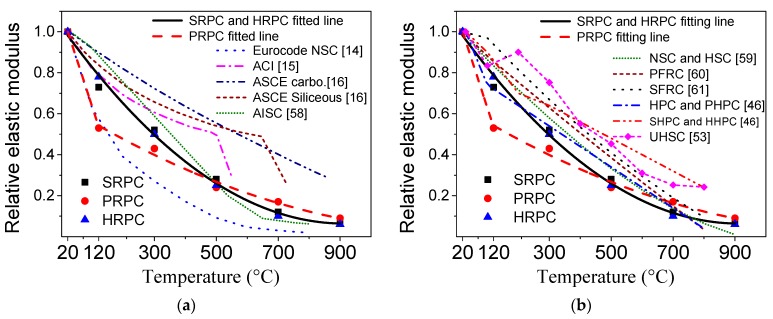
Comparison of the normalized elastic modulus of RPC with (**a**) design codes and (**b**) previous research.

The ratio of elastic modulus at high temperature to corresponding unheated elastic modulus has been plotted along with those obtained from the design codes [14,15,16,58] and relevant literature [46,53,59,60,61] in Figure 9. It is evident that the elastic modulus obtained from Eurocode-2 [14] is comparable to that of PRPC up to 120 °C but at higher temperatures, it is unconservative. Furthermore, the elastic moduli as per ACI [15], ASCE [16], and AISC [58] design recommendations are unconservative for RPC. The elastic modulus of RPC is at the lower bound of the earlier research. This might be due to the build-up of vapor pressure and increasing incompatibilities between steel fibers and RPC matrix at high temperature.

The formulas for elastic modulus were derived from the experimental results by regression analysis. The formula for normalized elastic modulus for SRPC and HRPC is given in Equation (9), whereas for PRPC are given in Equation (10).

(9) $$E_{m}^{T}/E_{m}=1.023-0.0021T+1.152\times 10^{-6}T^{2},\;20\;{}^{\circ}C\leq T\leq 900\;{}^{\circ}C$$

(10) $$E_{m}^{T}/E_{m}=\{\begin{array}{ll} 1.094-0.00465T, & 20\;{}^{\circ}C\leq T\leq 120\;{}^{\circ}C\\ 0.65-9.44\times 10^{-4}T+3.56\times 10^{-7}T^{2}, & \;120\;{}^{\circ}C<T\leq 900\;{}^{\circ}C \end{array}$$

where $E_{m}^{T}/E_{m}$ is the normalized elastic modulus, $E_{m}^{T}$ is the elastic modulus at high temperature, $E_{m}$ is unheated elastic modulus, and T is the temperature in degree Celsius.

#### 3.1.5. Peak Strain

**Figure 10:**
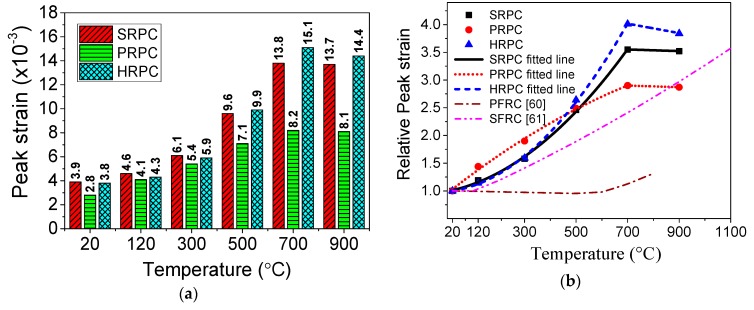
Peak strain of RPC: (**a**) absolute values and (**b**) normalized values.

The strain corresponds to the maximum stresses is defined as peak strain in this study. The peak strain of RPC is shown in Figure 10. The peak strain of SRPC, PRPC, and HRPC at original unheated condition is 3.9 × 10^−3^, 2.8 × 10^−3^, and 3.8 × 10^−3^, respectively. The peak strain of all types of RPC was gradually increasing up to 700 °C. However, above 700 °C, it remained unchanged. The peak strain at 900 °C for SRPC, PRPC, and HRPC was 3.52, 2.87, and 3.84 times their values at room temperature. The effect of PP fibers was not significant as compared to steel and hybrid fibers on peak strain at high temperature. Since PP fibers melt down at 167 °C, the resultant microcracks have no significant role in decreasing the stiffness of RPC. The Peak strain of RPC is greater than PFRC and SFRC. This increase in peak strain is attributed to the thermal incompatibilities between cement past, ITZ, and steel fibers at high temperature [29]. Furthermore, the decomposition of CH and C-S-H gel, quartz phase transformation, micro-crack, and macro-crack development at high-temperature result in an increase in peak strain of RPC [70,71]. 

The formulas for peak strain were derived from the experimental results by regression analysis. The formula for normalized peak strain for SRPC, PRPC, and HRPC are given in Equations (11)–(13), respectively.

(11) $$\varepsilon_{c}^{T}/\varepsilon_{c}=\{\begin{array}{ll} 0.99+9.18\times 10^{-4}T+3.92\times 10^{-6}T^{2}, & \;20\;{}^{\circ}C<T\leq 700\;{}^{\circ}C\\ 3.65-1.49\times 10^{-4}T, & 700\;{}^{\circ}C<T\leq 900\;{}^{\circ}C \end{array}$$

(12) $$\varepsilon_{c}^{T}/\varepsilon_{c}=\{\begin{array}{ll} 0.96+0.0037T-1.286\times 10^{-6}T^{2}, & \;20\;{}^{\circ}C<T\leq 700\;{}^{\circ}C\\ 3.005-1.49\times 10^{-4}T, & 700\;{}^{\circ}C<T\leq 900\;{}^{\circ}C \end{array}$$

(13) $$\varepsilon_{c}^{T}/\varepsilon_{c}=\{\begin{array}{ll} 0.99+4.79\times 10^{-4}T+5.5\times 10^{-6}T^{2}, & \;20\;{}^{\circ}C<T\leq 700\;{}^{\circ}C\\ 4.6-8.42\times 10^{-4}T, & 700\;{}^{\circ}C<T\leq 900\;{}^{\circ}C \end{array}$$

where $\varepsilon_{c}^{T}/\varepsilon_{c}$ is the normalized peak strain, $\varepsilon_{c}^{T}$ is the peak strain at high temperature, $\varepsilon_{c}$ is unheated peak strain, and *T* is the temperature in degree Celsius.

#### 3.1.6. Stress-Strain Curve

**Figure 11:**
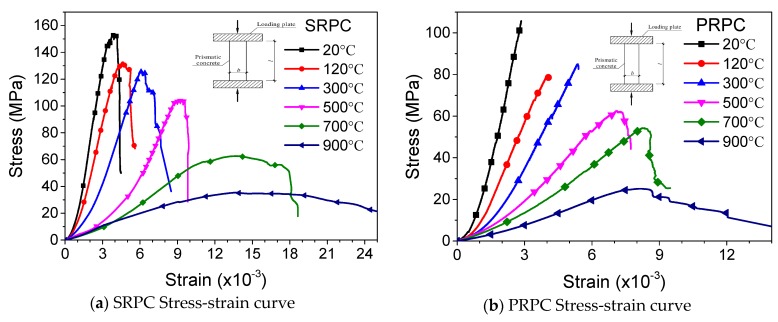

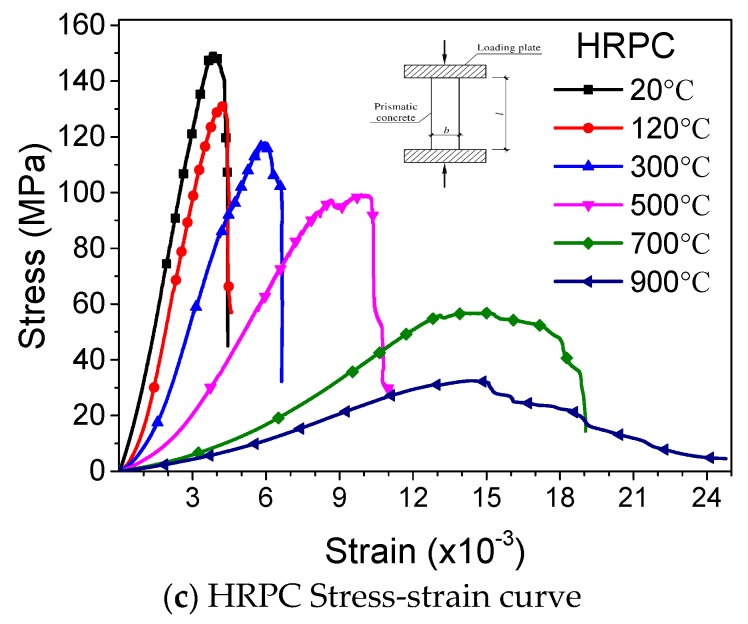
Stress-strain curve of RPC at high temperature.

The mechanical response and fire resistance of concrete structures mostly depend on stress-strain curves. The stress-strain curves of SRPC, PRPC, and HRPC are plotted in Figure 11. It can be seen that SRPC and HRPC have a ductile behavior and the descending portions are also measured during the testing. Whereas the PRPC have a quite brittle behavior up to 300 °C, however further heating makes the microstructure porous and it becomes ductile too. With increasing temperature, the compressive strength was diminished, the peak strain was increased and the elastic modulus was decreased. The curves become flattered above 500 °C. This is due to the strength loss and increasing peak strain. It is obvious from Figure 11 that the stress-strain curves were initially linear, trailed by a parabolic section up to maximum load and before failure a descending portion was obtained, except for PRPC at lower temperature.

**Figure 12:**
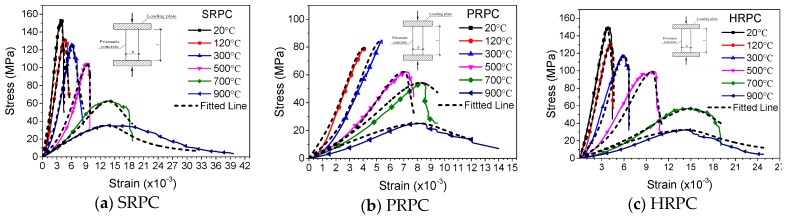
Comparison of the proposed model with experimental results.

The test data were modeled and the residual stress-strain model of Zheng et al. [30] has been used by redefining the model parameters through regression analysis of the test data. The equation developed can be used for the fire-resistance analysis of RPC structures in computer programs. The ascending portion of the stress-strain curve is controlled by parameter *α* and the descending portion by *β*. The values of *α* are defined by the ratio of high-temperature elastic modulus to the ambient temperature elastic modulus. The parameter *β* is controlled by the area under the descending portion of the curve. By regression analysis of the test data presented in Figure 11, the parameters *α* and *β* were computed and are presented in Table 3. The constitutive model developed is given in Equation (14). The model result has been plotted along with the experimental data for easy reference in Figure 12.

(14) $$y=\{\begin{array}{ll} \alpha x+(5-4\alpha )x^{4}+(3\alpha -4)x^{5}, & \;0\;\leq x\leq 1,\\ \frac{x}{\beta {\left(x-1\right)}^{2}+x}\;, & \;x\geq 1. \end{array}$$

where $x=\frac{\varepsilon }{\varepsilon_{c,T}}$; $y=\frac{\sigma }{f_{c,T}}$; *α* and *β* are equations parameters whose values are given in Table 3.

### 3.2. RPC Behavior in Real-Life Building Fire

Knowledge about the behavior of concrete in real life building fire is very important. Concrete protects the reinforcement steel from the direct exposure of fire. For fire-resistance design, the cover thickness recommended in the design codes are more than normal conditions. Since concrete thermal conductivity is smaller, it protects the steel temperature from being raised to critical conditions. Recently, the co-authors have conducted full-scale fire resistance test of reinforced RPC beams [72]. The tests were conducted following ISO834 standard fire time-temperature curve [45]. This time-temperature exposure is more realistic with building natural fire, especially with wooden furniture [73]. The hybrid combination of 0.2% PP and 2% steel fibers were used in the preparation of RPC. The composition of the materials was the same as that of HRPC in this study. Furthermore, the beams were wrapped in fire insulation materials. The structural responses, crack patterns, and fire endurance were measured and recorded during the fire tests. The results show that the RPC strength and stiffness were not severely deteriorated and the fire endurance was more than 2 h. With increasing cover thickness from 25 mm to 35 mm, the fire endurance can increase up to 34.2% of the former beam. The mid-span deflection was within the limit as the RPC material did not deteriorate and the rebars were not softened because of slower increase in their temperature. The hybrid combination of fibers and insulation were very effective in preventing the fire-induced spalling and to increase the fire endurance of RPC structural members.

### 3.3. Microstructure

#### 3.3.1. TG and DSC Analyses

**Figure 13:**
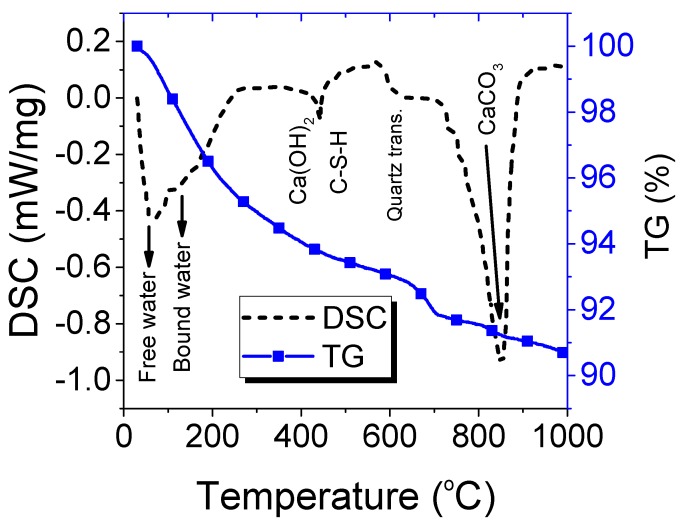
Thermogravimetric (TG) and differential scanning calorimetry (DSC) curves for RPC at different temperatures.

The TG and DSC tests were performed to evaluate the physical and chemical changes at high temperature in the RPC matrix. The results are shown in Figure 13. These changes provide the exact technical support for the interpretation of the results of mechanical properties at high temperature. The slope of TG was very steep up to 300 °C, this indicates the evaporation of free water and chemically bound water (gel water). The mass loss of 4% out of total 9.35% occurred in this stage. The gradient of TG curve was decreasing with further increase in temperature above 300 °C. The decomposition of C-S-H and CH hydrates starts above 300 °C. A steep drop was observed near 700 °C, which represents the decomposition of calcite. The results of DSC tests are more obvious and major dips were observed at different temperatures. A main dip exists in the vicinity of 150 °C, which indicates the evaporation of free water and chemically bound water (gel water). The dip at around 500 °C verifies the decomposition of CH and C-S-H hydrates. The quartz phase transformation can also be seen in a small drop near 600 °C, whereas a big dip around 700 °C–900 °C shows the decarbonation of calcite (CaCO_3_).

#### 3.3.2. Mercury Intrusion Porosity

**Figure 14:**
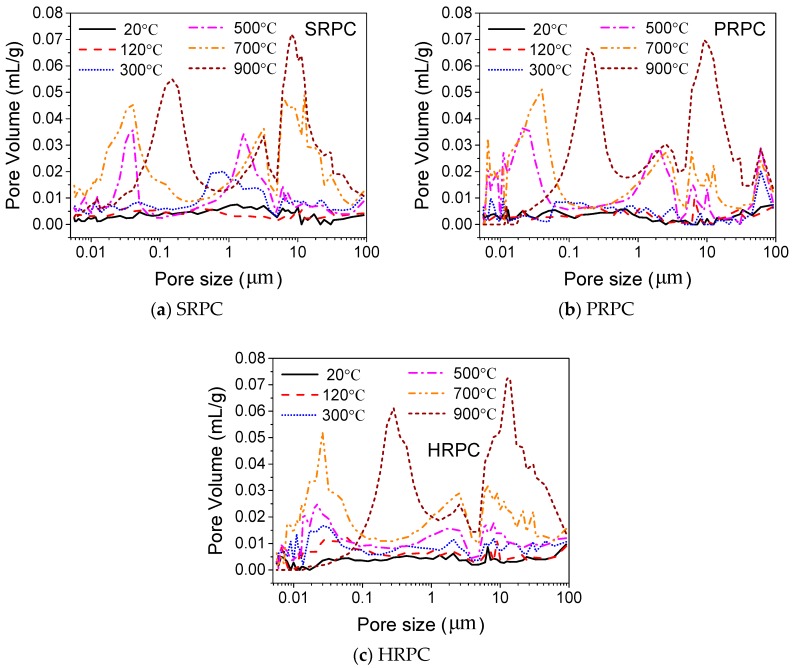
Changes of pore size distribution at high temperature (**a**) steel fiber reinforced RPC (SRPC), (**b**) polypropylene fiber reinforced RPC (PRPC), and (**c**) hybrid fiber reinforced RPC (HRPC).

**Figure 15:**
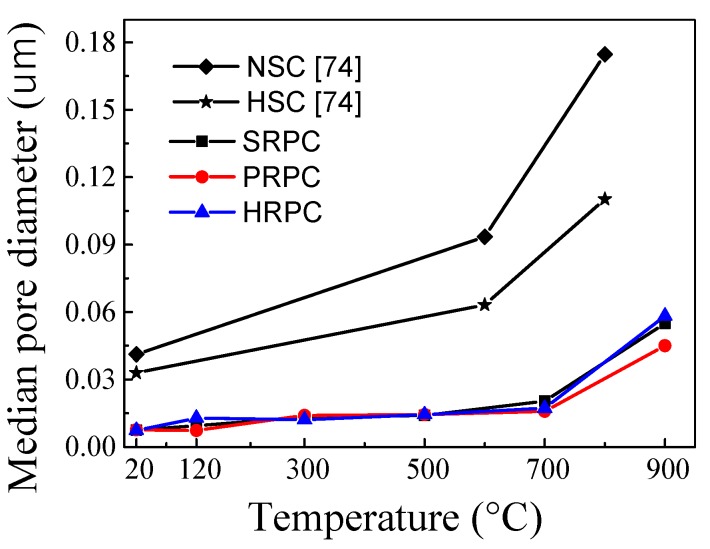
Median pore diameters of different RPCs at high temperature.

**Figure 16:**
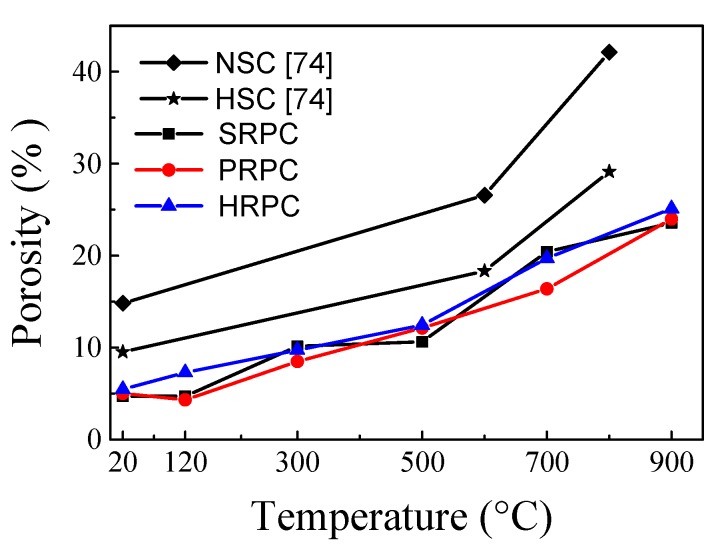
Porosity of different RPCs at high temperature.

The pore size distribution, pore diameter, and porosity of RPC were measured using MIP as shown in Figure 14, Figure 15 and Figure 16, respectively. The pore volume increased with increasing temperature. The pore volume for the target temperatures lower than 700 °C increased in the range of 0.01~0.1 μm and above 1 μm. However, for 900 °C, it has maximum volume in the range of 5~15 μm. The higher porous volume is attributed to the physical and chemical changes that occurred during high temperature as explained in the succeeding section.

The median pore diameter of RPC had no significant change up to 500 °C. Furthermore, the porosity and median pore diameter were not affected by different types and composition of fibers. The median pore diameter of SRPC, PRPC, and HRPC at 20 °C is 7.5 × 10^−3^, 7.6 × 10^−3^, and 7.4 × 10^−3^ μm, respectively. Whereas at 900 °C it becomes 7.33, 5.22, and 7.89 times their median pore diameters at the unheated condition. This shows that steel fibers cause excessive thermal incompatibilities at high temperature and the resultant pore diameter is high. The porosity of SRPC, PRPC, and HRPC at room temperature was 4.69%, 5%, and 5.45%, respectively. The increase in porosity at 900 °C is 5.01, 4.79, and 4.60 times the ambient temperature porosity of SRPC, PRPC, and HRPC, respectively. The porosity of all three types of RPC was gradually increased with increasing temperature. This might be due to the evaporation of free water and bound water, decomposition of C-S-H and CH hydrates, and microcrack development due to uneven expansion between cement paste and aggregates [71]. Furthermore, a comparison has been made among the median pore diameter and porosity of RPC with NSC and HSC [74]. It is obvious that RPC has lower median pore diameter and porosity than NSC and HSC at all target temperatures.

#### 3.3.3. XRD Patterns

**Figure 17:**
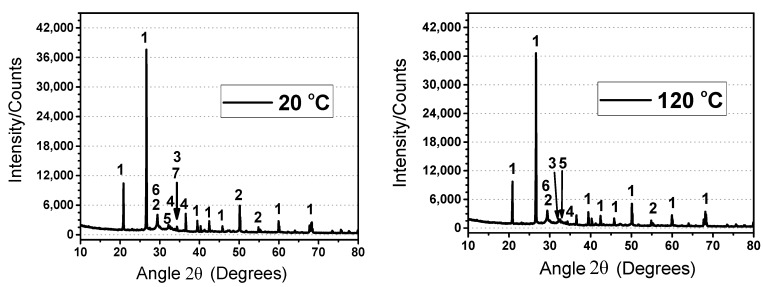

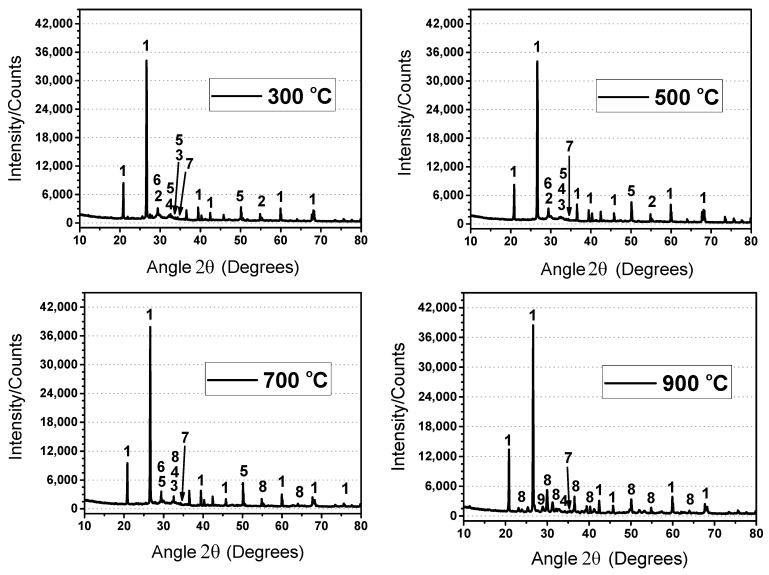
**X-**ray diffraction (XRD) patterns of RPC at different temperatures. Where: 1 >> Silica (SiO_2_); 2 >> C-S-H gel; 3 >> C_3_A; 4 >> C_2_S; 5 >> C_3_S; 6 >> CaCO_3_; 7 >> CH; 8 >> β-CaSiO_3_ (Wollastonite), 9 >> CaO.

The XRD pattern of RPC after exposure to the studied target temperatures are given in Figure 17. Since the basic composition and raw materials are the same, the only difference was the type of fibers that does not contribute to the hydration reaction. Therefore, the XRD analysis of the plain RPC was performed. Quartz sand is a major component of RPC which is evident from the various peaks at different phase angles (2θ). The main peaks of quartz were observed around 20.85° and 26.63°. It was observed that these peaks were reduced at 120 °C and 300 °C. However, above 300 °C, a gradual increase in the height of peaks was observed. The reduction might be due to the hydration reaction of the pozzolanic effect, whereas the increase in the height of peaks at the higher temperature might be due to the decomposition of C-S-H hydrates [75]. The quartz under normal temperature is in trigonal form, which is also called α-quartz. However, at 573 °C, it converts to hexagonal form (β-quartz). Furthermore, heating changes it into hexagonal β-tridymite at 870 °C [76]. However, this transformation was not observed at 700 and 900 °C, because it is a reversible transformation. The predominant hydrates C-S-H, CH, C_3_A, C_2_S, C_3_S, and calcite were identified within 25° to 35°. It is evident that the peaks of C-S-H, CH, and C_3_A were reduced gradually at and above 500 °C. The decomposition of C-S-H started above 500 °C and its peaks were reduced. However, an obvious peak of β-CaSiO_3_ (Wollastonite) was overserved abundantly at 900 °C, which is a decomposed form of C-S-H gel. Furthermore, the peaks of C_2_S, and C_3_S increased above 500 °C due to the decomposition of C-S-H gel. The peak of CaO was also observed at 900 °C, which was obtained due to the decomposition of CaCO_3_.

#### 3.3.4. SEM and EDX Analyses

**Figure 18:**
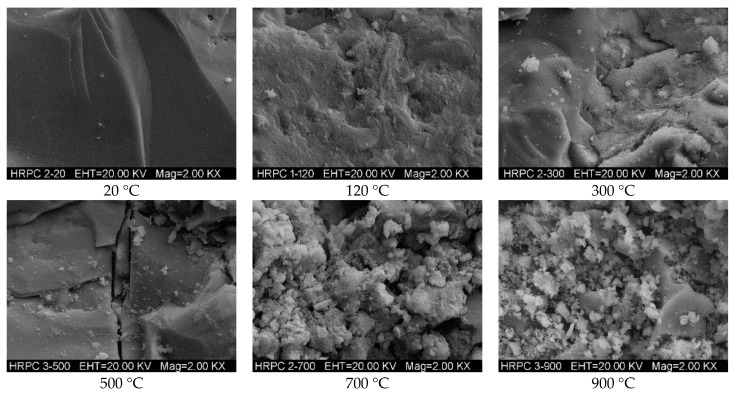
SEM micrographs of RPC matrix at different temperatures.

**Figure 19:**
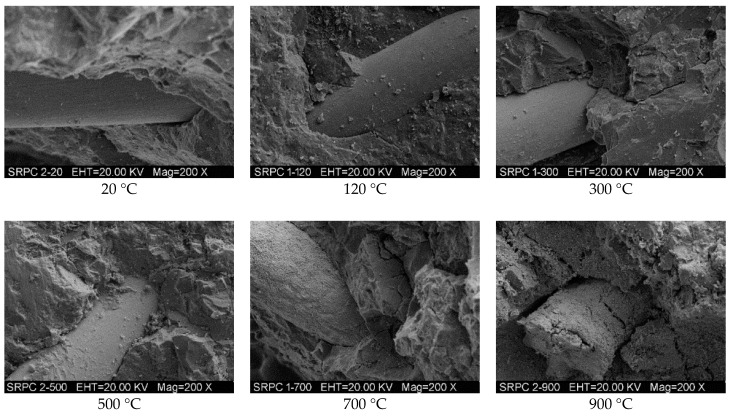
SEM micrographs of the bonding interface between steel fiber and RPC matrix at different temperatures.

**Figure 20:**
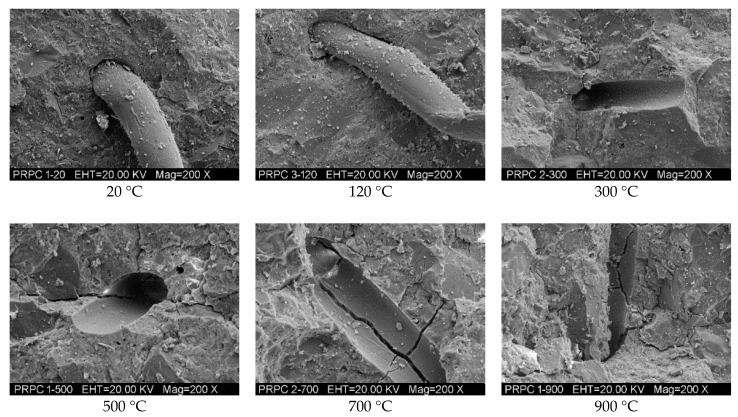
SEM micrographs of the bonding interface between PP fiber and RPC matrix at different temperatures.

The microstructure of RPC has been examined using SEM after exposure to different target temperatures. The macro level fluctuating pattern of mechanical properties has been assessed from the SEM micrographs at the micro level. Figure 18 shows the micrographs of RPC matrix, whereas the bond interface of steel fibers-matrix and that of PP fibers-matrix are shown in Figure 19 and Figure 20, respectively. The micrographs show that RPC has a very dense microstructure at ambient temperature; furthermore, a strong bond exists between fibers and matrix (Figure 18, Figure 19 and Figure 20). At 120 °C, the rough surface is developed because of the expansion of water and vapors between the C-S-H gel layers, which also decreases the binding forces [77]. At 300 °C, despite a few microcracks, the RPC matrix is more even and smoothly covered by the hydration products. This is because silica fume reacts with cement hydrates and produces additional C-S-H hydrates. Moreover, the quartz and SO_2_ present in silica fume work as a catalyst and accelerate the hydration reaction.

PP fibers are melted at 167 °C and the micro-channels left behind decreases the stress concentration due to releasing vapor pressure, thus increasing the strength of RPC. Whereas the steel fibers remain closely knit with the RPC matrix and no obvious cracks develop at this stage. The decomposition of CH and C-S-H hydrates from 400–500 °C makes the RPC structure weaker and porous, as can be seen in Figure 18. The cracks become obvious and strength starts to decrease at this stage—the steel fiber and cement interface becomes weaker and the crack width increases. At 700 °C, the cement hydrates become dissolved, the quartz transformation occurs and the whole surface gets covered with rough, grainy, and rose bush type structures. At 900 °C, the layered plates melt down and the number of cracks and pores increases significantly. The entire bonding interface is converted into a bushy rough surface. The steel fibers are also burned down and many microcracks appear on it. The bonding interface of steel fibers with cement matrix is totally broken.

**Figure 21:**
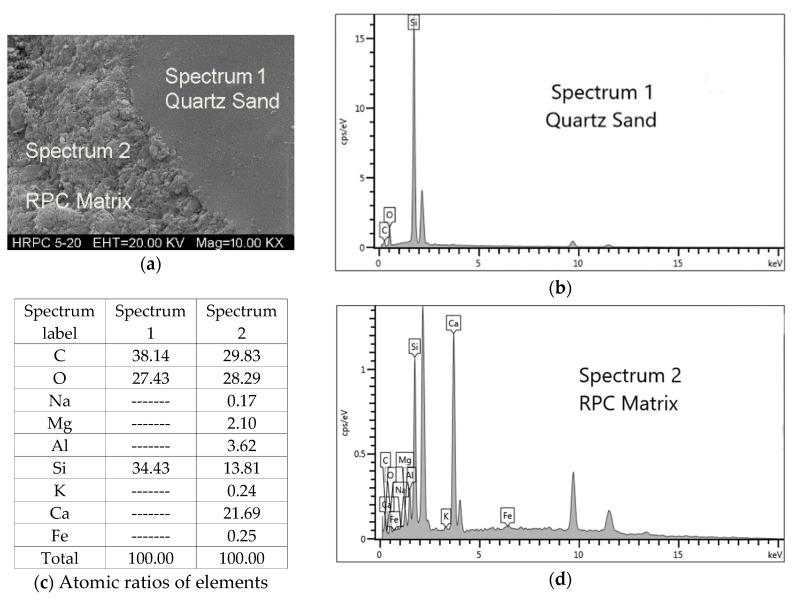
SEM micrographs of the bonding interface between quartz sand and RPC matrix at room temperature.

**Figure 22:**
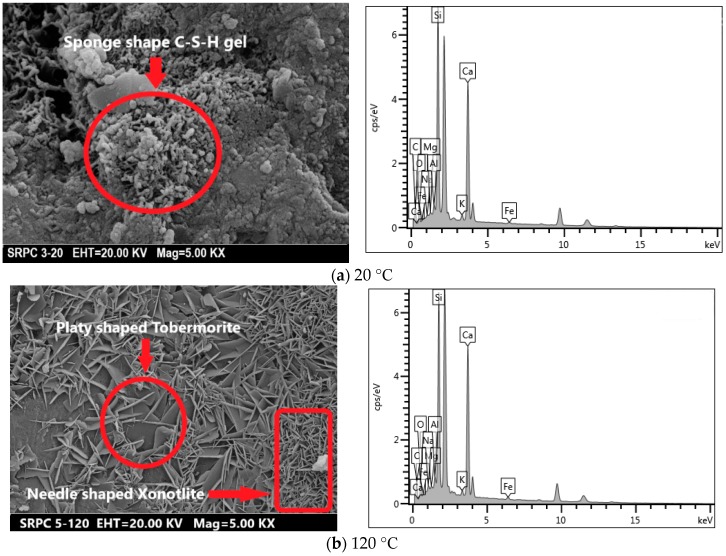

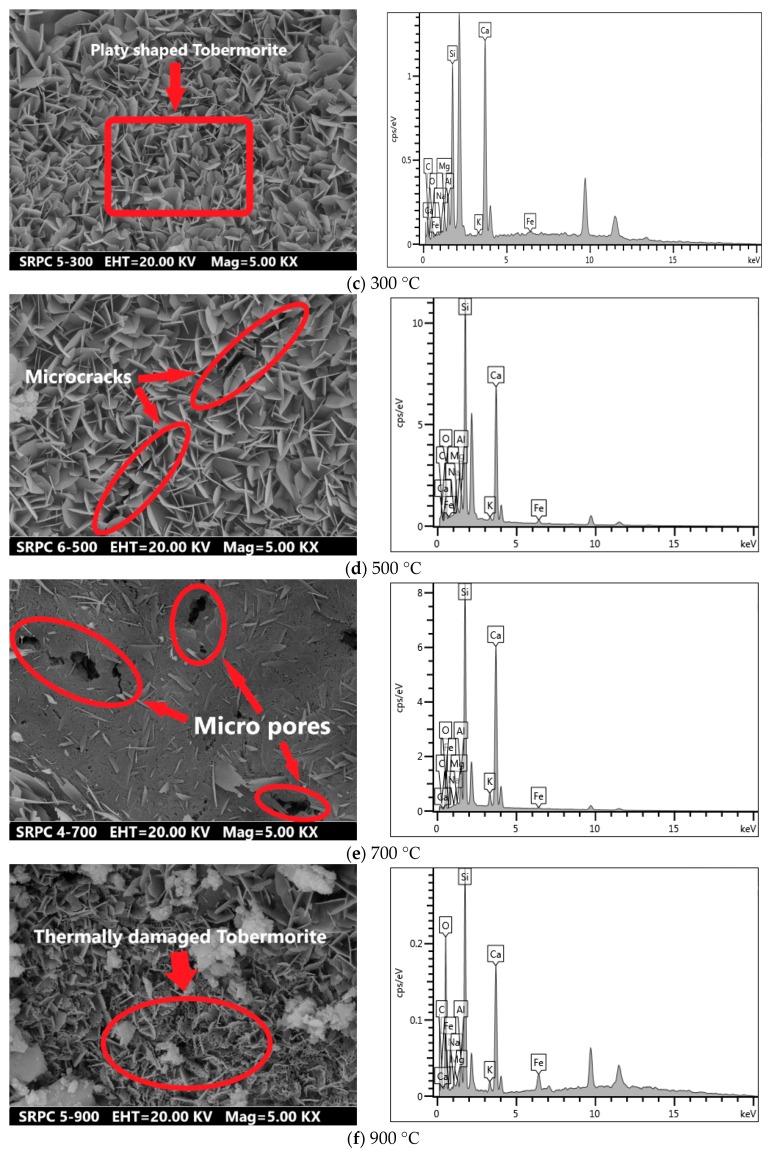
SEM micrographs of secondary hydrates and energy dispersive **X**-ray spectroscopy (EDX) analysis of RPC matrix at different target temperatures.

The ITZ between the quartz sand and cement paste of unheated RPC is shown in Figure 21. The result was also verified by EDX analysis. There were no obvious cracks found in the ITZ area of unheated specimens, as evident from Figure 21. The quartz-paste bond was tightened and the quartz boundary was blurred in the paste. The close packing effect is attributed to the sequential hydration reactions of the smaller size grains of silica fume during the steam curing process. Furthermore, the heating in oven causing internal autoclave curing effect results in the hydration of the leftover particles of cementitious materials [7]. This contributes to the improved durability and mechanical performance of RPC. The increase in crack size in the ITZ area can be examined indirectly from the median pore diameter analysis, as shown in Figure 15 [78]. There is no obvious change in the median pore diameter of RPC up to 500 °C. Therefore, the size of cracks in the ITZ area is not affected severely up to 500 °C. However, above 500 °C, the median pore diameter increases significantly. This shows that the crack width and the number of cracks in the ITZ area are also increased. Furthermore above 500 °C, the entire bonding interface was converted into a bushy, rough, and grainy surface (Figure 18), which makes it difficult to measure the crack width accurately. The EDX tests were performed on the RPC matrix far away from the quartz aggregate, which is shown in Figure 22. The atomic ratios measured during EDX were used for the classification of different types of hydrate based on the following equations [79].

(15) For C-S-H,    0.8≤Ca/Si≤2.5; (Al+Fe)/Ca≤0.2

(16) For CH,    Ca/Si≥10; (Al+Fe)/Ca≤0.4; S/Ca≤0.04

The sponge-like structures are found in unheated specimens. The EDX analysis shows that these are C-S-H gel. After exposure to 120 °C, some needle shape xonotlite (Ca_6_Si_6_O_17_(OH)_2_) and platy-shaped tobermorite (Ca_5_Si_6_O_16_(OH).5H_2_O) were found inside the micro-pores. They are responsible for the lower porous volume and lower porosity of RPC. The EDX analysis shows that the Ca/Si ratio for the needle shape structures was 1 whereas that for platy shape-structures was 0.83, which is the molar ratio for xonotlite and tobermorite, respectively. The plate-shaped tobermorite was also seen at 300 °C. The microcracks developed at 500 °C and further heating up to 700 °C produced micropores in the RPC matrix. Heating up to 900 °C caused severe damage to the existing tobermorite hydrates, as seen in Figure 22f.

## 4. Conclusions

The following conclusions were obtained from the experimental results:

- The compressive strength of PRPC is significantly lower than those of SRPC and HRPC due to the lower elastic modulus and lower strength of PP fibers as compared to steel fibers. The compressive strength started to decrease at 120 °C, however at 300 °C, a partial recovery was seen for all types of RPC. Above 300 °C, a gradual decrease in cubic and a sharp decrease in prismatic strength were observed.
- The compressive strength of RPCs below 300 °C is lower than that obtained from the design codes. However, above 300 °C, the strength retention is much higher than those of the design codes. The recession in strength was more than those of NSC, HSC, PFRC, and SFRC, except for HPC and UHPC, up to 300 °C. This is mainly because of the coupled effect of vapor pressure and loading at high temperature. However, above 300 °C, RPC performs better than the traditional types of concrete due to its superior microstructure and effective role of fibers.
- PRPC has the lowest split-tensile strength and flexural strength as compared with SRPC and HRPC. The HRPC split-tensile strength and flexural strength are higher than those of the SRPC at ambient temperature due to the additional resistance provided by the PP fibers against the tension force. The degradation of split-tensile strength for all types of RPC is gradual with increasing temperature. PRPC performance is poor when compared with the design recommendations and earlier research. However, the strength reduction was less in SRPC and HRPC due to the combined effect of superior microstructure and fibers.
- The elastic modulus has been severely degraded with increasing temperature. The peak strain of all types of RPC gradually increased up to 700 °C, while it remained unchanged after 700 °C. SRPC and HRPC have ductile behavior; however, PRPC was quite brittle below 300 °C, while further heating above 300 °C makes the microstructure porous and it becomes ductile too.
- The predominant hydrates C-S-H, CH, C_3_A, C_2_S, C_3_S, and calcite were identified within 25 to 35 ° from XRD analysis. The decomposition phase of the main hydrates (C-S-H gel and Calcium hydroxide) started above 500 °C, which causes reduction in the strength. The peaks of C_2_S, C_3_S, and calcite were increased gradually above 500 °C. The wollastonite was overserved abundantly at 700 and 900 °C, which is a decomposed form of C-S-H gel.
- Generally, the porosity of RPC was gradually increasing with increasing temperature. Moreover, RPC has lower median pore diameter and porosity than the NSC and HSC at all target temperatures. The median pore diameter of RPCs has no significant change up to 500 °C; however, it increases sharply above 700 °C.
- The microstructure study through SEM and EDX analyses reveals the presence of secondary hydration products such as xonotlite and tobermorite in the pores of RPC. It can be concluded that RPC possesses very dense and crystallized structure up to 300 °C. However, from 500 to 900 °C, the strength recession starts due to the development of obvious microcracks, decomposition of cement hydrates, and weakened bonds between the steel fibers and RPC matrix.
- It can be said that HRPC is a promising material for structures with a high risk of fire due to its non-explosive behavior and lower strength recession.

## Figures and Tables

**Table 1:** Chemical compositions of cement, silica fume, and slag in percentage.

Cementitious Materials	SiO_2_	Al_2_O_3_	Fe_2_O_3_	CaO	MgO
Cement	21.40	5.45	3.50	64.48	1.46
Silica fume	94.50	0.50	0.45	0.60	0.70
Slag	34.90	14.66	1.36	37.57	9.13

**Table 2:** Mix proportions for steel fiber reinforced RPC (SRPC), polypropylene fiber reinforced RPC (PRPC), and hybrid fiber reinforced (HRPC).

Constituents	SRPC	PRPC	HPRPC
Ordinary Portland cement (kg/m^3^)	800.53	816.42	815.18
Silica fume (kg/m^3^)	240.16	244.33	245.31
Slag (kg/m^3^)	120.08	122.16	120.08
Quartz coarse sand (kg/m^3^)	480.32	490.32	480.32
Quartz fine sand (kg/m^3^)	480.32	490.32	480.32
Water reducer (kg/m^3^)	34.82	35.47	35.40
PP fiber (kg/m^3^)	-----	2.73 (0.3% ^a^)	1.82 (0.2% ^a^)
Steel fiber (kg/m^3^)	157 (2% ^a^)	-----	157 (2% ^a^)
Water (kg/m^3^)	185.72	189.20	188.81
w/b ratio	0.16	0.16	0.16
Workability (mm)	175	180	170

^a^ Steel and PP fibers are measured as % of concrete volume.

**Table 3:** Equation parameters at different temperatures.

Temperature (°C)	Parameters	
	*α*	*β*
20	0.77	18.78
120	0.75	26.26
300	0.52	12.23
500	0.51	118.30
700	0.82	7.47
900	1.27	5.62
